# Gut microbiota modulation in gastrointestinal disorders: current evidence and therapeutic perspectives

**DOI:** 10.3389/fcimb.2025.1740322

**Published:** 2026-01-05

**Authors:** Meng-Ying Zhang, Shao-Yu Chen, Yu-Hua Lin, Xing-Xing Yuan

**Affiliations:** 1 Department of Intervention, Shanghai Xuhui Central Hospital, Shanghai, China; 2 Department of Emergency Medical, Shanghai Baoshan District Hospital of Integrated Traditional Chinese and Western Medicine, Shanghai, China; 3 Department of Respiratory Medicine, Xiamen Traditional Chinese Medicine (TCM) Hospital Affiliated to Fujian University of Traditional Chinese Medicine, Xiamen, Fujian, China; 4 Department of Respiratory Medicine, Xiamen Hospital, Dongzhimen Hospital, Beijing University of Chinese Medicine, Xiamen, Fujian, China; 5 Department of Gastroenterology, Heilongjiang Academy of Traditional Chinese Medicine, Harbin, China

**Keywords:** fecal microbiota transplantation, gastrointestinal disorders, gut microbiota, metabolites, short-chain fatty acids

## Abstract

Gut microbiome medicine is a promising field in functional medicine, offering personalized treatment strategies for gastrointestinal disorders. Advanced metagenomic and metabolomic technologies have revealed the gut microbiome’s systemic influence, extending to distant organs like the brain and lungs. While small molecules and genes facilitate these effects, the gut microbiota’s greatest abundance and activity are concentrated in the gastrointestinal tract, particularly in the distal regions. The balance of microbial communities in the small and large intestines is crucial for gastrointestinal health. However, the dominance of pathogenic bacteria can disrupt this balance, leading to tissue damage and contributing to gastrointestinal disorders. Emerging interventions, such as probiotics, fecal microbiota transplantation, and dietary enrichment with short-chain fatty acids, show potential in restoring microbial balance, enhancing immune function, and potentially protecting against carcinogenesis. Current evidence from clinical trials and animal models supports the therapeutic role of gut microbiome modulation in reversing gastrointestinal disorders. However, variability in study outcomes highlights the need for further research to standardize these approaches for clinical practice. This review underscores the gut microbiome’s pivotal role in gastrointestinal health and the therapeutic promise of functional medicine in addressing these disorders. This review also explores emerging interventions, such as phage therapy and engineered microbes, and provides comparative analyses of microbiota signatures and therapeutic approaches across different gastrointestinal disorders.

## 1 Introduction

The gut microbiota (GM) comprises a vast and diverse community of microorganisms, including bacteria, viruses, fungi, and archaea, residing in the human gastrointestinal tract. This complex ecosystem performs numerous essential functions for the host, including immune system regulation, defense against pathogens, and metabolism of dietary nutrients, thereby profoundly influencing overall health (Cryan, 2025; Sun and Xu, 2025). A stable and diverse GM is crucial for maintaining gastrointestinal homeostasis, supporting processes such as digestion, nutrient absorption, and waste excretion. Beneficial members of the GM stimulate immune function, facilitate cell regeneration, and synthesize vital compounds like enzymes, vitamin K, and biotin (Yang et al., 2024; Wang Q. et al., 2025).

This equilibrium, however, is fragile and can be disrupted by a state known as dysbiosis. Dysbiosis refers to an imbalance in the gut microbial community structure, often characterized by a loss of beneficial microbes, an overgrowth of potentially harmful pathobionts, and/or a reduction in overall microbial diversity. Multiple factors can precipitate dysbiosis, including dietary patterns, pharmaceutical use (especially antibiotics), chronic physiological and psychological stress, infections, and host genetics (Lee and Lee, 2021; Ma et al., 2023; Böhm et al., 2025). Functionally, gut bacteria can be categorized based on their roles: immunomodulatory bacteria (*Faecalibacterium prausnitzii*), beneficial metabolite-producing bacteria (SCFA-producing *Roseburia* and *Lachnospiraceae*), barrier-maintaining bacteria, and pathobionts that can promote inflammation in a dysbiotic state.

The consequences of dysbiosis are far-reaching. Reduced gut microbial diversity has been strongly associated with a spectrum of autoimmune, metabolic, and chronic gastrointestinal disorders, including inflammatory bowel disease (IBD), irritable bowel syndrome (IBS), obesity, and diabetes (Wang et al., 2024a; Iliev et al., 2025; Li et al., 2025; Liu et al., 2025). Dysbiosis can compromise intestinal barrier integrity, leading to increased permeability (“leaky gut”), aberrant immune activation, and sustained inflammation, which are key drivers in the pathogenesis of many gastrointestinal diseases.

To counteract dysbiosis and restore a healthy microbial balance, several therapeutic strategies have been developed. Probiotics are live microorganisms that, when administered in adequate amounts, confer a health benefit on the host. Prebiotics are selectively fermented ingredients that allow specific changes in the composition and/or activity of the gastrointestinal microbiota, conferring benefits upon host health. Their mechanisms, often mediated through the promotion of SCFA-producing bacteria, are complex and can be influenced by an individual’s baseline microbiota. By serving as a substrate for these commensals, prebiotics promote the production of health-promoting metabolites like short-chain fatty acids (SCFAs), enhance gut barrier function, and inhibit the colonization of pathogens. However, the mechanisms of prebiotics are complex and can be influenced by an individual’s baseline microbiota, and their efficacy varies across different clinical contexts.

When dysbiosis is severe or resistant to simpler interventions, more direct approaches are needed. Fecal microbiota transplantation (FMT) represents a powerful therapeutic modality that aims to restore a healthy GM by transferring processed fecal material from a healthy, carefully screened donor into a recipient’s gastrointestinal tract (Cao et al., 2025). The rationale for FMT is rooted in the concept of “re-booting” the microbial ecosystem: by introducing a complete, diverse, and stable community of microbes, it can outcompete pathobionts, re-establish metabolic functions, and correct the immune dysregulation characteristic of dysbiosis. While FMT has demonstrated remarkable efficacy, primarily in treating recurrent *Clostridium difficile* infection (CDI) where it resets the microbiota following antibiotic disruption (Lavelle and Sokol, 2022), its application in other gastrointestinal disorders is actively being investigated.

**Figure 1:**
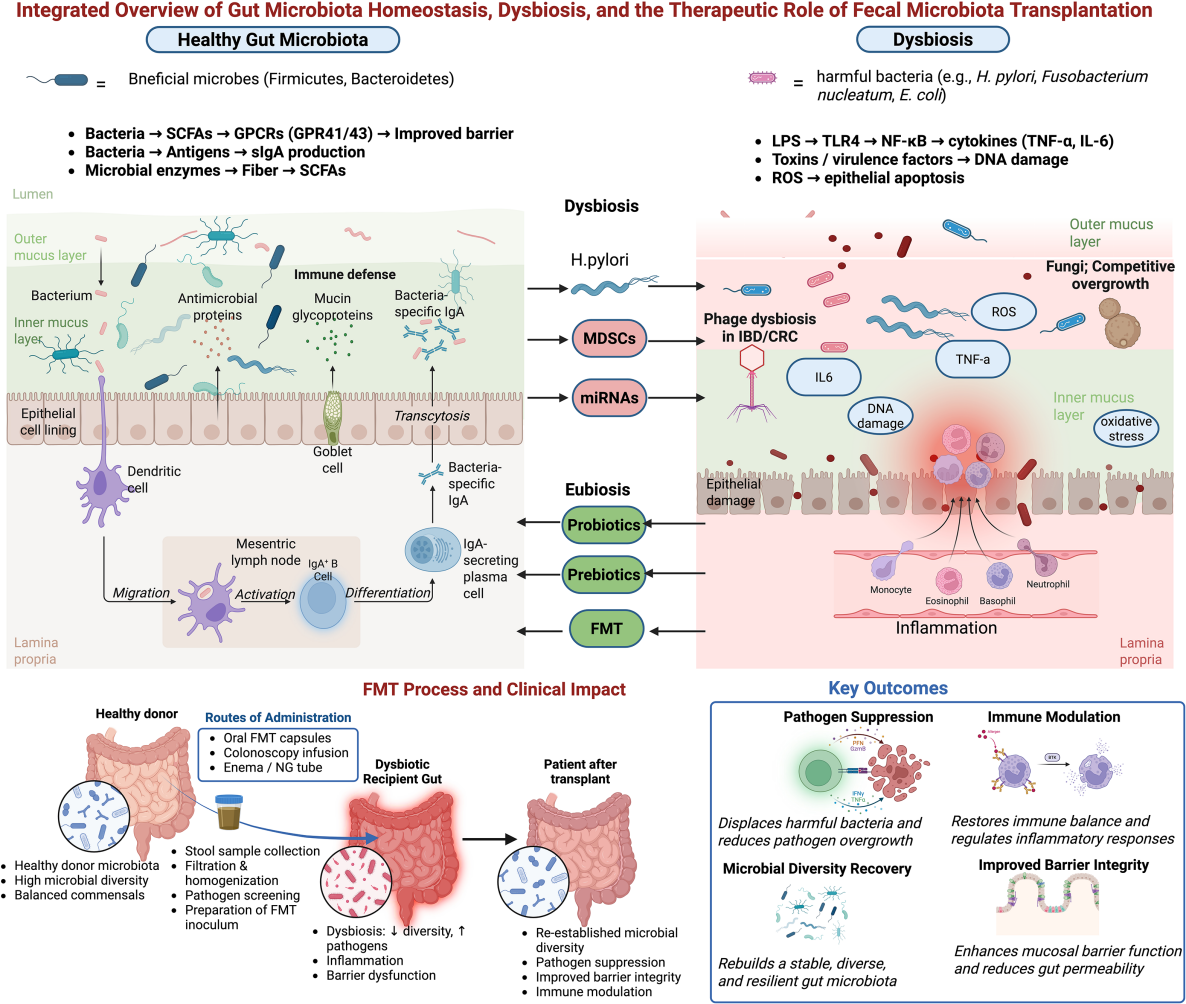
Gut microbiota physiology in health and gastrointestinal disorders, and the therapeutic role of fecal microbiota transplantation. The left panel represents a healthy gut environment characterized by beneficial microbial communities, SCFA production, epithelial integrity, immune tolerance, and mucosal defense mechanisms. In contrast, the right panel depicts dysbiosis driven by harmful taxa, which disrupt the mucus layer, increase oxidative stress, elevate pro-inflammatory cytokines, and contribute to epithelial damage, DNA injury, and chronic inflammation. The lower panel presents the FMT process and its clinical relevance. Key therapeutic outcomes include pathogen suppression, immune modulation, microbial diversity recovery, and enhancement of mucosal barrier function benefits demonstrated most robustly in recurrent *Clostridioides difficile* infection and increasingly supported in inflammatory bowel disease (IBD) and other dysbiosis-associated gastrointestinal disorders.

Despite these advances, significant challenges remain. The precise mechanisms underlying dysbiosis development are still being unraveled, and it is often difficult to distinguish whether observed microbial changes are a cause or a consequence of disease. Methodological limitations in microbial characterization and the high degree of interindividual variability also complicate research and clinical translation (Shen et al., 2025). A comprehensive understanding of the molecular interactions within the GM and between the GM and the host is essential for developing effective, targeted therapies. Therefore, this review aims to systematically characterize GM imbalances associated with major gastrointestinal disorders, examine their functional consequences, and critically evaluate the current evidence and future potential of microbiome-based therapeutic interventions, including prebiotics, probiotics, and FMT (**Figure 1**).

## 2 Gut microbiota in gastrointestinal disorders

### 2.1 Colorectal cancer

The increased prevalence of pathobionts in the gut correlates not only with lipopolysaccharide diffusion but also with the expression of various virulence factors, ultimately contributing to carcinoma development. Contemporary research approaches cancer development from novel perspectives, though bacterial and viral infections remain associated with approximately 15% of cancer cases. Viral pathogens initiate tumorigenesis through inflammatory processes, tumor growth stimulation, and host genome integration of active oncogenes that promote immunosuppression (Xiao et al., 2025). GM alterations mediated by virulence factors, particularly through β-catenin signaling, can stimulate excessive growth of both normal and adenoma cells. The specific virulence factor Fusobacterium adhesin A facilitates this process while simultaneously promoting the proliferation of *Fusobacterium nucleatum*, a microorganism associated with increased epithelial permeability and microbial invasion. Such compositional shifts in intestinal bacteria also introduce the carcinogenic and genotoxic potential of *Fusobacteria* phylum members (Jin et al., 2024).

The scientific understanding of CRC has evolved from focusing on specific bacterial associations to recognizing broader GM dysbiosis patterns. Current evidence strongly suggests the adenoma-cancer cascade correlates with elevated *Fusobacterium* prevalence. Experimental manipulation of these microbial populations using metronidazole demonstrated reduced colon tumor growth in murine models, indicating potential biomarker applications for CRC (Wang et al., 2023). Beyond *Fusobacterium*, multiple bacterial species including *Lactobacillus* spp., *Streptococcus bovis*, *Porphyromonas* spp., and *Roseburia* spp. show associations with polyp size progression. Additionally, CRC patients exhibit decreased *Firmicutes* levels in both tumor biopsies and stool samples, though the magnitude of this decrease varies across studies and populations. While these observations require further validation, the diminished production of SCFAs, resulting from reduced *Faecalibacterium* and *Roseburia* populations during CRC development, leads to butyrate deficiency. Consequently, the gastrointestinal environment loses crucial anti-carcinogenic properties, including the induction of tumoral cell apoptosis, T-cell mediation capacity, and proliferation inhibition mechanisms (Ma et al., 2022).

### 2.2 Clostridium difficile infection

Patients with CDI consistently demonstrate elevated *Proteobacteria* levels alongside reduced *Bacteroidetes* and *Firmicutes* phyla populations (Spigaglia, 2024). While the mere presence of toxigenic *Clostridium difficile* in the host proves insufficient as an intestinal inflammation biomarker, the *TcdA* and *TcdB* enzymes that are secreted during the vegetative growth phase substantially compromise cytoskeleton integrity (Paparella et al., 2021). These glycosyltransferase enzymes modify cytoplasmic Rho GTPases, with the secreted toxins serving as primary pathogenic agents that initiate intestinal tract infections (Paparella et al., 2022). Moreover, antibiotics targeting *Clostridium difficile* paradoxically promote *Enterobacteriaceae* proliferation while diminishing *Lachnospiraceae* abundance, as evidenced by animal model studies. Although prematurely declaring *Clostridium difficile* gut expansion as a biomarker for *Enterobacteriaceae*-dominated microbiota would be speculative, this phenomenon has been documented in elderly human subjects. CDI patients admitted to intensive care units exhibit characteristic microbial shifts: reduced diversity in *Cryptomycota*, *Deferribacteres*, and *Acetothermia* taxa, along with decreased *Saccharomycetes* and *Clostridia* genera, contrasted by overgrowth of *Cryptomycota*, *Acetothermia*, and *Deferribacteres* (Wang et al., 2020). Mouse model studies utilizing *Clostridium difficile* strain VPI 10463 spores revealed microbial diversity improvements, particularly demonstrating positive correlation between *Akkermansia muciniphila* and *Bacteroides fragilis* treatment outcomes (Wu et al., 2022). Established CDI biomarkers include depleted populations of *Lachnospiraceae*, *Bacteroides*, and *Ruminococcaceae* (Berkell et al., 2021).

### 2.3 Necrotizing enterocolitis

The incidence of NEC in pediatric populations is promoted by immature immunity, enteral feeding practices, bottle-feeding, and resulting dysbiosis. This pathological condition primarily triggers gut inflammation in premature newborns, with particularly high susceptibility observed in infants weighing less than 1500g at birth, demonstrating a mortality rate approaching 30%. While various interventions have been explored to alleviate or eliminate NEC, several proposed mechanisms including breast milk lactoferrin and immunoglobulins, oral supplementation with prebiotics (galacto-oligosaccharide, lactose, and fructose-oligosaccharide), and probiotic *Bifidobacterium breve* BBG-001 - have proven ineffective. The precise etiology of NEC remains at preliminary stages of investigation. *Enterobacteriaceae* associated with NEC produce hexacylated lipopolysaccharides that function as potent pyrogens, inducing inflammatory responses mediated through TLR4 signaling pathways (Gomart et al., 2021). While most research has focused on extrauterine factors, emerging evidence suggests intrauterine factors such as antenatal antibiotic exposure may also confer NEC risk (Klerk et al., 2022).

The GM of low-birth-weight preterm infants appears linked to neurodevelopmental impairment through dysbiosis-mediated mechanisms involving the gut-brain axis. This connection is increasingly recognized as a potential diagnostic marker for NEC alongside late-onset sepsis (Duan et al., 2020). The gut-brain axis facilitates bidirectional communication, and in NEC, systemic inflammation and microbial metabolites may impact the developing brain, though the precise molecular pathways require further elucidation. The primary microbial determinant of NEC in preterm infants appears to be intestinal *Gammaproteobacteria*, which suppress *Bifidobacterium* populations that would normally dominate in breastfed term infants. Prolonged antibiotic use, known to increase NEC risk, likely exacerbates this reduction in bifidobacterial counts. Furthermore, activation of TLR4 pathways appears to contribute to the suppression of *Bifidobacterium* colonization in preterm neonates (Huang et al., 2025).

### 2.4 Inflammatory bowel disease

Among chronic gastrointestinal disorders, IBD, encompassing Crohn’s disease (CD) and ulcerative colitis (UC), remains poorly understood etiologically. Recent investigations have associated IBD pathogenesis with GM alterations and modifications in tumor necrosis factor and interleukin signaling pathways (Xie et al., 2025). IBD patients typically exhibit reduced *Firmicutes* alongside elevated *Proteobacteria* and *Actinobacteria* populations. Notably, studies in genetically susceptible mice indicate dysbiosis may precede overt inflammation. *Firmicutes prausnitzii*, a bacterial species with important anti-inflammatory properties and significant SCFA production capacity, demonstrates particularly low abundance in IBD patients. This reduction is especially pronounced in CD cases. In contrast, *Enterococcus*, *Lactobacillus*, and *Bifidobacterium* species may be dominant in certain IBD patient cohorts (Tamburini et al., 2024).

Current evidence regarding microbiota dysbiosis patterns in IBD subtypes remains inconsistent, necessitating further research before clinical applications can be considered (Santana et al., 2022). For instance, while CD correlates with generally low *Bacteroides* levels, UC dysbiosis specifically associates with increased *Bacteroides fragilis* and *Bacteroides vulgatus* populations (Nomura et al., 2021; Tharu et al., 2024). Other studies report *Bifidobacterium* and *Lactobacillus* dominance in UC groups, contrasted by *Fusobacterium* and *Escherichia-Shigella* predominance in CD cohorts (Zhang et al., 2020; Scanu et al., 2024). *E. coli* Nissle 1917, an alternative probiotic to mesalazine for UC treatment, exemplifies this by outcompeting other *E. coli* strains for iron during inflammation (Zhao et al., 2022; Wang Y. et al., 2025).

### 2.5 Gastric cancer

Research has established the oncogenic properties of *Helicobacter pylori* (*H. pylori*), demonstrating its association with malignant transformation in gastric tissues. Both normal and pathogenic *H. pylori* strains increase adenocarcinoma risk by inducing chronic inflammation and disrupting β-catenin signaling in epithelial cells (Wang et al., 2024b). Herbal formulations demonstrate significant effects on GM by enhancing postoperative gastrointestinal function recovery, improving tumor response, and conferring better performance status while reducing adverse effects (Xu et al., 2022). The established pathogenesis of gastric cancer involves disordered innate and adaptive immunity, imbalanced GM colonization, mucosal barrier dysfunction, genetic variations, and environmental and personalized risk factors. Chinese herbal products such as Xiaoyaosan modulate GM composition by increasing *Lactobacillus*, *Proteus*, and *Bacteroides* abundance while reducing *Rickerella* and *Desulfovibrio*, serving as biomarkers for gastric environment modulation (Zhao et al., 2021). The adenoma-carcinoma sequence in gastric carcinogenesis typically shows reduced anti-inflammatory bacteria and SCFA-producing bacteria alongside increased pro-inflammatory species. Bacterial-induced cytotoxic effects causing DNA damage have been well documented. However, non-invasive screening methods based on specific microbiota markers remain necessary for clinical applications (Wu et al., 2024).

Beyond *H. pylori*, the most prevalent bacterial pathogen associated with GC reduced gastric acidity caused by *H. pylori* enable colonization by other potentially carcinogenic microbiota. Certain bacteria thriving in hypoacidic conditions, including *Lactobacillus*, *Neisseria*, *E. coli*, and *Staphylococcus*, produce carcinogenic N-nitroso compounds through nitrogen compound conversion. Notably, *Lactobacillus*-mediated increases in gastric lactic acid concentration promote inflammation while providing energy for tumor angiogenesis (McDermott et al., 2000). *H. pylori* induces damage through dysregulation of DNA transcription factors and reactive nitrogen intermediate generation, triggering inflammatory cascades in gastric mucosa (Nadeem et al., 2020). Studies in Chinese populations identified *Dialister pneumosintes*, *Peptostreptococcus stomatis*, *Parvimonas micra*, *Streptococcus anginosus*, and *Slackia exigua* as significantly enriched bacterial taxa in GC cases (Yang et al., 2020). Korean population studies identified *H. pylori*, *Propionibacterium acnes*, and *P. copri* as the strongest GC risk factors, while *Lactococcus lactis* appeared protective (Leonard et al., 2020). Animal models of GC showing *Proteobacteria* and *Actinobacteria* abundance corroborate findings in human gastric carcinogenesis (Coker et al., 2018).

### 2.6 Irritable bowel syndrome and bacteriocins

The well-established association between gastroenteritis events and IBS confirms that gut dysbiosis leading to abnormal intestinal immune activation and subsequent inflammation represents a significant risk factor. Fermentable oligo-, di-, and monosaccharides and polyols reduce luminal bacteria populations (*Bifidobacterium* and *Faecalibacterium prausnitzii*) while controlling dysbiosis at lower concentrations, though their long-term microbiome effects require further investigation. Despite conflicting study results, IBS patients consistently demonstrate increased *Firmicutes* and decreased *Bacteroidetes* (Pittayanon et al., 2019; Napolitano et al., 2023).

Bacteriocins, which are toxic proteins and peptides secreted by gut bacteria, represent a competitive survival mechanism targeting rival taxa for nutrient and biomolecule utilization. These secretions include short antimicrobial peptides called microcins. Bacteriocin-expressed immunity proteins provide protection against toxic effects within the producing bacterial populations (Milajerdi et al., 2021).

Bacteriocin production is well-documented in Enterobacteriaceae, such as colicins in *E. coli* and pesticins in *Yersinia pestis*, often expressed under nutrient stress (Darbandi et al., 2022). Their activity can exacerbate microbial population shifts during inflammation by inducing genotoxic and oxidative stress in susceptible bacteria. Beyond these, other forms like *Pseudomonas* pyocins and mechanisms in Gram-positive bacteria, such as *Enterococcus faecalis*, utilize diverse strategies like nucleic acid cleavage or plasmid-borne competition (Virolle et al., 2020) (**Table 1**).

**Table 1:** The role of gut microbiota in gastrointestinal disorders and interventions.

Gastrointestinal disorder	Key findings	Role and significance of gut microbiota	Reference
CRC	Herbal formula Xiao-Chai-Hu-Tang (XCHT) increased *Parabacteroides*, *Blautia*, and *Ruminococcaceae*.	Modulating the GM with XCHT downregulated the TLR4/MyD88/NF-κB pathway, inhibiting tumor growth and improving systemic inflammation in a depression-comorbidity model.	(Shao et al., 2021)
CRC	Prebiotics (fructooligosaccharides, xylooligosaccharides, etc.) increased *Bifidobacterium* and *Enterococcus* and decreased *Bacteroides*.	Prebiotic intake in perioperative CRC patients improved serum immunologic indicators (IgG, transferrin), suggesting GM modulation can support immune function during cancer treatment.	(Xie et al., 2019)
IBS	A randomized trial showed increased *Clostridia* and serum bile acid marker 7α-hydroxy-4-cholesten-3-one (C4).	A *Clostridia*-rich microbiota enhances bile acid excretion in diarrhea-predominant IBS-D, directly linking a specific microbial shift to a key disease mechanism.	(Zhao et al., 2020)
IBS/Functional Dyspepsia	Ginger root powder increased *Actinobacteria*, *Parabacteroides*, and *Bacillus*, and decreased *Blautia*.	Ginger supplementation altered gastrointestinal bacteria composition, which was correlated with improved indigestion symptoms, highlighting GM as a therapeutic target for functional disorders.	(Crichton et al., 2023)
Chronic Constipation	Psyllium husk supplementation altered GM diversity and specific OTUs.	The alleviation of constipation symptoms by psyllium was associated with changes in the gut microbiota, underscoring the role of microbial metabolism in gut motility.	(Yang et al., 2021)
IBD and Hepatic Encephalopathy	Rifaximin-α reduced mucin-degrading species (*Streptococcus*, *Veillonella*, *Akkermansia*, *Hungatella*).	The antibiotic’s efficacy is linked to its ability to remodel the gut microbiota, promoting a less inflammatory environment and aiding gut barrier repair.	(Patel et al., 2022)
SIBO	A simple sugar diet decreased small intestinal microbial diversity and increased permeability.	Diets triggering functional gastrointestinal symptoms directly cause microbial dysbiosis and impaired barrier function in the small intestine, confirming the role of GM in symptom generation.	(Saffouri et al., 2019)
Drug-Induced Dysbiosis	Proton Pump Inhibitor (PPI) use increased gut abundance of the oral bacterium *Streptococcus anginosus*.	PPIs alter the gut microenvironment, permitting the translocation and survival of oral bacteria in the gut, a novel mechanism for PPI-related dysbiosis.	(Xiao et al., 2024)
CDI	FMT led to a major rewiring of the microbial network.	FMT’s protective effect against recurrent infection is mediated by restoring a complex and competitive gut microbiota ecosystem, displacing pathogens.	(Rashidi et al., 2023)
Cancer Therapy Side Effects	A probiotic cocktail reduced the severity of oral mucositis.	Probiotics may protect against mucosal injury from chemo/radiotherapy by modulating local and systemic immune responses, though specific GM changes were not detailed.	(Xia et al., 2021)
Metabolic Health & CRC Risk	A Mediterranean and weight loss intervention targets the bile acid-gut microbiome axis.	This study design investigates how dietary modulation of the GM and its metabolic output (bile acids) can reduce the risk of colorectal cancer.	(McLeod et al., 2023)
General Gut Health	Walnut consumption increased *Faecalibacterium*, *Clostridium*, *Dialister*, and *Roseburia*.	Dietary walnuts enrich beneficial, SCFA-producing bacteria, suggesting a mechanism for their health benefits through positive GM modulation.	(Holscher et al., 2018)
Glucose Metabolism	Inulin-propionate ester (IPE) increased *Actinobacteria* and decreased *Clostridiales*.	Delivering propionate to the colon via IPE alters the microbiota profile and improves insulin sensitivity, linking a microbial metabolite to host metabolism.	(Chambers et al., 2019)
Detoxification	Probiotic yogurt increased *Blautia* and *Bifidobacterium* and decreased heavy metal levels.	Specific probiotics can enhance the gut microbiota’s capacity to bind and excrete toxic heavy metals, presenting a novel bioremediation approach.	(Feng et al., 2022)

### 2.7 Comparative overview of microbiota alterations

A comparative analysis of the gastrointestinal disorders discussed reveals a recurring pattern of gut microbiota dysbiosis, commonly characterized by a depletion of beneficial, SCFA-producing bacteria, often within the *Firmicutes* phylum, and a concomitant expansion of pro-inflammatory taxa, frequently *Proteobacteria*. This shared ecological disturbance underscores a fundamental breakdown in microbial homeostasis that predisposes the gastrointestinal tract to disease. However, this common backdrop gives rise to distinct, disorder-specific pathological mechanisms through unique “microbiota-molecule-host” interactions. For instance, carcinogenesis in CRC is significantly driven by *Fusobacterium nucleatum*-mediated activation of the β-catenin pathway, while the pathology of CDI is uniquely defined by direct toxin-induced damage. Similarly, NEC hinges on hyperactive TLR4 signaling in response to lipopolysaccharides, and IBD involves NF-κB pathway activation and a loss of immune tolerance.

These distinct mechanistic pathways, in turn, dictate the application of targeted therapeutic strategies. While FMT serves as a broad-spectrum intervention to reset the microbial community in CDI, other disorders require more nuanced approaches. These include phage therapy or prebiotics to target specific pathobionts like *F. nucleatumin* CRC, *H. pylori* and herbal formulations to modulate the carcinogenic microenvironment in gastric cancer, and biologic therapies or engineered microbes to counter specific immune dysregulation in IBD. Even in IBS, where dysbiosis is subtler, interventions like the low FODMAP diet aim to correct metabolically driven symptoms. Thus, the clinical management of gastrointestinal diseases is increasingly informed by an understanding of both the common themes of dysbiosis and the specific microbial drivers and mechanisms underlying each condition.

## 3 Therapeutic strategies of gut microbiota modulation in gastrointestinal disorders

The GM possesses the capacity to restore its normal microbial composition in response to various influencing factors. Notably, GM contributes to SCFAs production, which plays a crucial role in maintaining gastrointestinal health (Liu C. et al., 2024; Yuan et al., 2025). While SCFAs provide significant health benefits, consumption exceeding WHO recommendations may predispose individuals to pro-inflammatory conditions such as obesity (Xiong et al., 2022; Han et al., 2024). Current evidence suggests sex may represent a determinant factor in dietary response variability, with effects ranging from transient to long-lasting (Andrews et al., 2024). However, research confirms that no single factor alone proves sufficient to induce dysbiosis. The remarkable resilience and adaptability of GM to environmental changes, coupled with its diverse nutrient utilization capacity, enable prolonged resistance to dysbiotic states (Fassarella et al., 2021).

Commensal bacteria may transition into opportunistic pathogens following even minor compositional shifts, permitting pathological overgrowth of competing species. While initial research focused on major phyla fluctuations as dysbiosis indicators, contemporary understanding emphasizes the disproportionate impact of marginal pathogenic bacterial groups (Gong and Xin, 2021). In addition, SCFA metabolic byproducts demonstrate dual roles in inflammatory processes. Beyond their gut health benefits, these compounds’ anti-inflammatory properties support the clinical application of probiotic and prebiotic therapeutic strategies (He et al., 2020). Emerging interventions including FMT, probiotics, prebiotics, and synbiotics effectively eradicate pathogens while restoring healthy microbiota composition, thereby enhancing gut homeostasis and reinforcing intestinal barrier integrity. Despite minor side effects, FMT demonstrates approximately 90% efficacy for CDI treatment with favorable safety profiles (Karimi et al., 2024).

Probiotic administration confers multiple health benefits, including increased microbial diversity and pathogen growth inhibition (Kwoji et al., 2021; Maftei et al., 2024). Various probiotic strains exhibit mucosal adherence and competitive exclusion properties, with Bifidobacteria specifically producing digestive enzymes, vitamins, and demonstrating ammonia-reducing capabilities. Multi-strain probiotics such as Symprove not only generate SCFAs but also stimulate lactate production, modulate anti-inflammatory cytokines (IL-6, IL-10), and reduce pro-inflammatory mediators (MCP-1, IL-8) (Markowiak-Kopec and Slizewska, 2020). However, the efficacy of probiotics in conditions like IBD is variable, and specific recommendations regarding strain selection (*Lactobacillus* or *Bifidobacterium*-dominant formulations), dosage, and duration require further standardization through large-scale trials.

Prebiotic fibers derived from common vegetables promote intestinal colonization by *Bifidobacterium* and *Lactobacilli*, which help reduce intestinal permeability and mitigate metabolic endotoxemia. The efficacy of synbiotics (probiotic-prebiotic combinations) depends on dosage, clinical context, and specific strain characteristics (Parhi et al., 2024). Both anti-tumor and tumor-promoting mechanisms in the digestive tract are fundamentally mediated by microbial communities. Therapeutic strategies incorporating engineered anti-tumor approaches through GM modification and metabolite regulation show promise for restoring gut health (Lei et al., 2025). For example, GM-associated mesenchymal stem cell therapy for IBD demonstrates enhanced intestinal barrier integrity, immunoregulation, and upregulation of secondary bile acid biosynthesis, sphingolipid metabolism, and cellular regeneration pathways (Liu A. et al., 2024). Predictive GM markers related to gastrointestinal pathogenicity including intestinal repair mechanisms, innate immunity modulation, and barrier function alterations may help prevent ulceration and mucositis development (Shi et al., 2024).

Probiotic supplementation, dietary modification, and FMT represent gold-standard therapeutic approaches for maintaining gastrointestinal health. While primary outcomes focus on gut homeostasis, secondary benefits may include improved neurological, pulmonary, and endocrine function through GM modulation (Liu A. et al., 2024). Dietary inflammatory index scoring reveals that anti-inflammatory diets associate with reduced abundance of non-beneficial bacteria (*Prevotella stercorea*, *Veillonella rogosae*) while increasing butyrate producers (Zeb et al., 2025). Fungal cell walls components, particularly β-(1→3,1→6)-d-glucans, demonstrate immunomodulatory benefits when administered at optimal doses (Zuo et al., 2024). Beyond apoptosis induction, probiotics inhibit tumor growth through immune response modulation, proliferation control, and intestinal barrier reinforcement, while also exhibiting anti-carcinogenic activity via oxidative stress regulation (Mirhosseini et al., 2024). Mechanistically, probiotics suppress pro-inflammatory cytokine production, decrease intestinal permeability, reduce reactive oxygen species, and stimulate enterocyte proliferation through targeted signaling pathway inhibition (Vlassopoulou et al., 2021).

### 3.1 Emerging intervention technologies

Beyond conventional probiotics and prebiotics, several novel therapeutic strategies are under active investigation. Phage therapy offers a highly targeted approach to eliminate specific pathobionts. For instance, a 2025 clinical trial demonstrated the efficacy of a phage cocktail in selectively reducing Fusobacterium nucleatum loads in CRC patients, thereby remodeling the tumor microenvironment and enhancing response to chemotherapy (Ding et al., 2025; Wu et al., 2025).

Engineered microbes, or synthetic probiotics, represent a frontier in precision microbiome medicine. These are genetically modified bacterial chassis (*E. coli* Nissle 1917) designed to secrete therapeutic molecules, such as antimicrobial peptides (microcins) or immunomodulatory proteins, directly within the gut niche. While promising, their clinical translation requires rigorous safety assessments to address concerns regarding horizontal gene transfer and long-term ecological impact (Zhang et al., 2025).

Furthermore, the distinction between live probiotics and their inactivated counterparts, or postbiotics, is of growing clinical interest. Postbiotics, which include heat-killed microbes, cell-free supernatants, and purified microbial components (SCFAs, surface proteins), offer potential advantages in safety (no risk of translocation or antibiotic resistance gene transfer) and stability over live biotherapeutics. In IBD, certain postbiotic formulations have demonstrated efficacy comparable to live probiotics in inducing anti-inflammatory responses and enhancing barrier function, suggesting their utility in vulnerable patient populations (Calvanese et al., 2025).

## 4 Challenges and emerging directions

Despite promising findings, microbiome research faces significant challenges, including a lack of clinical reproducibility due to high inter individual variability influenced by geography, diet, and ethnicity. Furthermore, translating results from animal models to humans is complicated by physiological and microbiota differences. Safety and regulatory limitations, particularly for FMT regarding donor screening and standardization, also hinder clinical application (Cruz et al., 2020; Lima et al., 2020). Beyond established strategies, emerging areas show considerable promise. These include the role of GM in modulating immunotherapy outcomes for gastrointestinal cancers (Zhang et al., 2024), the potential of phage therapy to target specific pathogens (Herlo et al., 2024), the use of postbiotics (inanimate microorganisms and/or their components) (Huang et al., 2021), and the development of engineered microbial therapeutics (Mishra et al., 2025). While a detailed discussion is beyond this review’s scope, these avenues represent the frontier of GM-based interventions.

Although comprehensive systematic reviews have identified sample- and country-specific bacterial genera, larger multi-season cohort studies analyzing species-specific bacteria and their metabolites are needed to eliminate potential confounding factors (Muttiah and Law, 2025). Standardized testing protocols and GM metabolite efficacy require validation before clinical implementation (Zubair et al., 2024). For various solid tumors, GM profiles serve as modifiable biomarkers predicting immune checkpoint inhibitor adverse effects, supporting antibiotic reduction and dietary optimization for improved immunotherapy outcomes (Zhang et al., 2024). Current probiotic applications for chemotherapy-induced gastrointestinal complications primarily utilize *Bifidobacteria* and *Lactobacillus* strains, highlighting the need for more diverse probiotic formulations (He et al., 2023). SCFAs influence immunological, epigenetic, and molecular signaling pathways through the “food-microorganism-SCFAs” axis (Shuwen et al., 2019). Bacterial extracellular vesicles represent a novel therapeutic modality in oncology research (Moghaddam et al., 2025), with oral delivery applications showing particular promise for GM modulation, barrier enhancement, immune regulation, and tissue repair (Liu et al., 2023). These vesicles demonstrate diagnostic and therapeutic potential through immunomodulatory cargo delivery and inflammation modulation (Levy et al., 2024).

### 4.1 Personalized regulation pathways

To overcome the “one-size-fits-all” limitation, future therapies must move toward personalized GM modulation. This involves a multi-step pathway: First, baseline microbiota detection using 16S rRNA or shotgun metagenomic sequencing to establish key indicators like α/β-diversity and specific taxon abundances. Second, stratified intervention plans based on this profile; for example, IBD patients with a *Firmicutes* abundance below 30% might be prioritized for FMT, while those with high *Ruminococcaceae* could respond better to specific prebiotics. Finally, dynamic monitoring through repeated fecal metabolomics (tracking SCFA levels) or microbial sequencing is crucial for assessing response and adjusting therapy.

### 4.2 Gut microecological interactions

The gut ecosystem extends beyond bacteria to include fungi and viruses, which play critical roles in health and disease. Recent research underscores the significant role of fungal dysbiosis (mycobiota) in IBD pathogenesis, highlighting mechanisms such as immune activation via Dectin-1/CARD9/IL-17 pathways, fungal-bacterial interactions, and the potential of antifungal strategies and fungal-focused therapies (Chen et al., 2025). Fungi-bacteria interactions are increasingly implicated in GI disorders; for example, *Candida* overgrowth can exacerbate IBD by competing for nutrients with beneficial bacteria and directly stimulating pro-inflammatory responses (Ding et al., 2025). Conversely, phages (bacterial viruses) are key regulators of bacterial populations through targeted lysis of pathogens. An altered “virome” is noted in IBD and CRC, suggesting phages could be harnessed to precisely reshape the microbial community (Zhang et al., 2025). Beyond established strategies, emerging areas show considerable promise, including the role of GM in modulating immunotherapy outcomes for gastrointestinal cancers (Zuo et al., 2024), and the development of engineered microbial therapeutics and postbiotics, as discussed in Section 3.2.

## 5 Conclusion and future prospectives

The GM demonstrates remarkable resilience in maintaining its normal composition despite various influencing factors. Current evidence confirms that no single factor alone induces dysbiosis, as the microbiota’s inherent adaptability to environmental changes and diverse nutrient utilization capacity enable prolonged resistance to compositional shifts. However, commensal bacteria may transition into opportunistic pathogens following even minor microbial fluctuations, allowing pathological overgrowth of competing species. Early research focused on major phylum-level changes as indicators of dysbiosis, but contemporary understanding emphasizes the disproportionate impact of low-abundance pathogenic bacterial groups. A fundamental nutrient competition dynamic exists between host and microbial cells, where host utilization of microbial metabolites influences other gut microorganisms. Microbe-derived metabolites activate various sensors expressed in gut epithelia, with butyrate and propionate specifically stimulating proliferation of β-defensins, crucial microbiome regulators, through nuclear receptor activation, particularly peroxisome proliferator-activated receptors. These receptors additionally mediate anti-inflammatory effects that indirectly influence bacterial diversity.

This review has systematically outlined the associations between GM dysbiosis and major gastrointestinal disorders, highlighting both common and unique microbial signatures across conditions like IBD, IBS, CRC, and GC. It has also detailed the mechanisms and current evidence for various therapeutic strategies, including probiotics, prebiotics, FMT, and dietary interventions. Looking forward, several key challenges and research directions must be addressed to advance the field: Future efforts must focus on standardizing therapeutic protocols (FMT procedures, probiotic strains and dosages) and developing personalized GM modulation plans tailored to an individual’s baseline microbiota, disease subtype, and environmental context. There is a critical need to move beyond correlations and elucidate the precise molecular mechanisms by which specific microbes and their metabolites influence host physiology. This includes a deeper investigation of the gut-brain axis in disorders like NEC and the functional role of bacteriocins.

The significant variability in GM composition across populations and the limitations of animal models necessitate larger, more diverse human cohort studies that account for dietary, geographical, and genetic confounders. Future research should expand to study the complex interactions between gut bacteria, viruses (phages), fungi, and other members of the gut ecosystem, and how these communities collectively impact health and disease. The integration of emerging approaches, such as engineered microbes, postbiotics, phage therapy, and the manipulation of GM to improve cancer immunotherapy, holds great promise for developing next-generation therapeutics. By addressing these priorities, the field can overcome current limitations and fully realize the potential of GM modulation as a cornerstone of personalized medicine for gastrointestinal disorders.
